# Eustachian Valve-Enhanced Paradoxical Cerebral Embolism: A Case Report

**DOI:** 10.7759/cureus.47263

**Published:** 2023-10-18

**Authors:** Kei Nozue, Hajime Ikenouchi, Tatsuo Miyamoto, Naoki Yamamoto, Kaoru Endo

**Affiliations:** 1 Neurology, Sendai City Hospital, Sendai, JPN

**Keywords:** cryptogenic stroke, implantable loop recorder, acute myeloid leukemia (aml), persistent eustachian valve, paradoxical cerebral embolism

## Abstract

Cryptogenic stroke includes many suspicious embolic causes that do not fulfill the Trial of Org 10172 in Acute Stroke Treatment (TOAST) classification criteria. Atrial fibrillation (AF) is one of the major hidden causes of cryptogenic stroke, and an implantable loop recorder (ILR) is widely used for detecting AF. Herein, we report a case of paradoxical cerebral embolism due to a large Eustachian valve with large PFO under no molecular complete remission (CR) of acute monocytic leukemia (AMoL). A 75-year-old man arrived at our emergency room because of aphasia and right hemiparesis. He had a history of two cryptogenic strokes and implanted ILR. Magnetic resonance imaging showed left middle cerebral artery occlusion with slight acute ischemic lesion. The red clot was retrieved by mechanical thrombectomy, and complete recanalization was achieved. We checked ILR, but there was no AF. Transesophageal echocardiography revealed a large patent foramen ovale (PFO) and the large Eustachian valve in the right atrium. Although obvious deep vein thrombosis (DVT) was not detected in venous ultrasonography of the lower extremities, Wilms’ tumor 1 messenger ribonucleic acid (WT1mRNA) expression level was high, and AMoL was considered to be not in molecular CR, suggesting a high risk of thrombosis to the large Eustachian valve. From large PFO and no molecular CR of AMoL, we diagnosed him with paradoxical cerebral embolism. Ruling out of AF by ILR and other etiologies, such as aortic or carotid atherosclerosis and pulmonary shunt, also supported the diagnosis of paradoxical cerebral embolism. Even in the absence of obvious DVT, paradoxical cerebral embolism should be considered in cases of a large Eustachian valve and PFO with a hypercoagulable state.

## Introduction

Cryptogenic stroke includes many suspicious embolic sources that do not fulfill the criteria of cardioembolic stroke or other causes of stroke in the classification of Trial of Org 10172 in Acute Stroke Treatment (TOAST) [1], such as slight aortic atherosclerosis, carotid plaque, or pulmonary shunt. Although paradoxical cerebral embolism is usually diagnosed by deep vein thrombosis (DVT) and right-to-left shunt (RLS), there are some cases of paradoxical cerebral embolism without typical DVT [2]. Herein, we report a case of paradoxical cerebral embolism due to a large Eustachian valve with patent foramen ovale (PFO) and a hypercoagulable state due to acute monocytic leukemia (AMoL), which diagnosis was enhanced by the exclusion of AF by implantable loop recorder (ILR).

## Case presentation

A 75-year-old man visited the emergency department of our hospital with aphasia and right hemiparesis. He had a history of two ischemic stroke events four years before and two months before admission. In the first ischemic stroke, he showed the left posterior cerebral artery territory infarction. He was diagnosed with AMoL just before the onset of the first ischemic stroke, and he was treated by direct oral anticoagulant (DOAC) as a diagnosis of cancer-related stroke. As for AMoL, he was treated with chemotherapy and achieved clinical complete remission (CR). Thereafter, he was followed by a hematologist with Wilms’ tumor 1 messenger ribonucleic acid (WT1mRNA) expression level, a marker of residual disease of AML [3]. Although he was asymptomatic, WT1mRNA expression level had been consistently higher between 500 and 1000 copies/μgRNA (reference value: <50 copies/μgRNA), suggesting no molecular CR. DOAC was continued for two years and discontinued because of no stroke recurrence and maintained clinical CR of AMoL. In the second ischemic stroke event, he showed an embolic stroke in the left middle cerebral artery territory. In the workup for embolic sources, there were also no stenoses in major arteries; no high risk of cardioembolism such as old myocardial infarction, sick-sinus syndrome, and valvular heart disease; and other causes of stroke such as arterial dissection, aortic plaque, an active solid tumor, or pulmonary shunt in whole-body computed tomography. Although TEE revealed a large PFO, there was no DVT in his lower extremities. Since atrial fibrillation (AF) could not be ruled out, we implanted ILR for the detection of AF and started aspirin as secondary prevention. 

**Figure 1:**
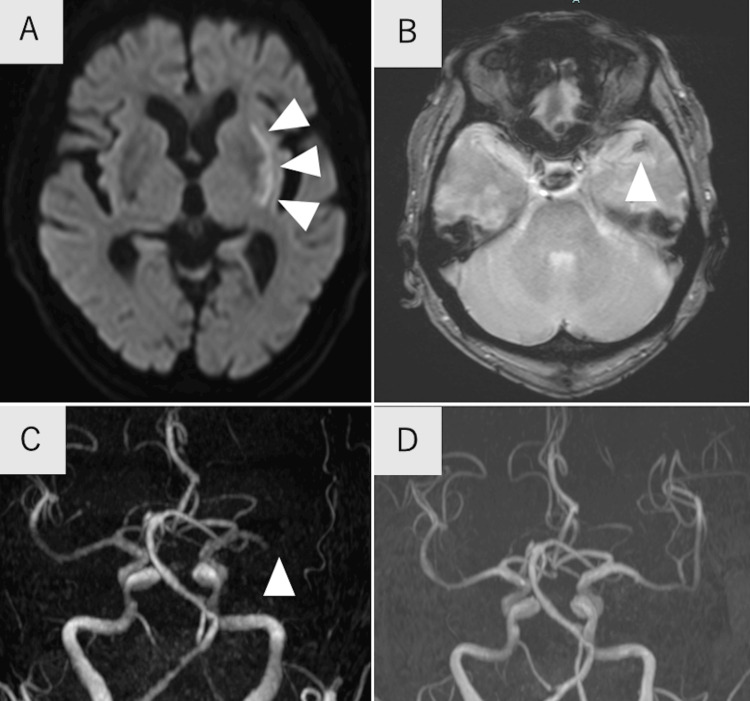
Imaging findings of the recurrent ischemic stroke A: DWI showed a high-intensity lesion in the left putamen (arrowheads). B: T2*-weighted imaging showed a susceptibility vessel sign in the left middle cerebral artery (arrowhead). C: MRA showed left M1 occlusion (arrowhead). D: MRA showed recanalization of the left middle cerebral artery after mechanical thrombectomy. DWI, diffusion-weighted imaging; MRA, magnetic resonance angiography

On arrival at the emergency room, neurological examination showed a Glasgow Coma Scale of 14 (E4V4M6), dysarthria, motor aphasia, and right hemiparesis with the National Institute of Health Stroke Scale (NIHSS) subscore of 2 in each upper and lower extremities, and the total NIHSS score was 7. However, his aphasia and right hemiparesis deteriorate and right hemispatial neglect emerged during magnetic resonance imaging (MRI), and his NIHSS score worsened to 20 after MRI. Diffusion-weighted imaging showed a high-intensity lesion in the left putamen (Figure 1A). T2*-weighted imaging showed a double-layered susceptibility vessel sign in the corresponding region to the left M1 (Figure 1B), and a magnetic resonance angiography showed left M1 occlusion (Figure 1C). By mechanical thrombectomy, complete recanalization was achieved (Figure 1D), and a red clot was retrieved.

**Figure 2:**
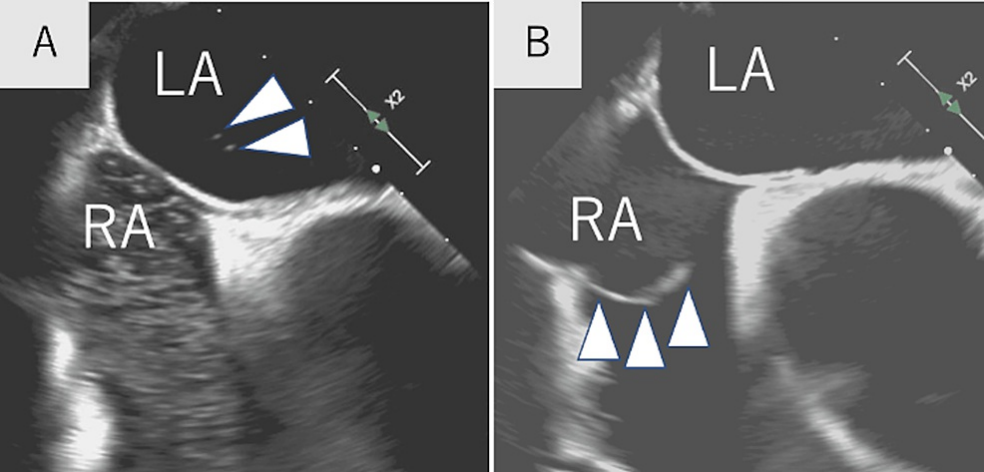
RLS and a large Eustachian valve on transesophageal echocardiography A: Microbubble test showed RLS (arrowheads). B: A large Eustachian valve was found in the right atrium (arrowheads). LA, left atrium; RA, right atrium; RLS, right-to-left shunt

As a recurrence of cerebral embolism, we checked ILR, but there were no embolic sources, including AF. Repeated transesophageal echocardiography showed multiple microbubbles in the left atrium both in rest and in the Valsalva maneuver, which suggested a large PFO (Figure 2A). TEE also revealed the large Eustachian valve in the right atrium (Figure 2B). 

There was also no DVT in his lower extremities. The blood test showed no signs of an abnormal coagulation state, such as an elevated D-dimer level, thrombotic predisposition, and autoimmune diseases. However, the WT1mRNA expression level was high (1300 copies/μg RNA), suggesting no CR of AMoL (Table 1).

**Table 1:** Laboratory findings of the present case Laboratory test results showed mild thrombocytopenia and elevated WT1mRNA expression level. There was no thrombotic predisposition or abnormal coagulation state. The additional blood tests for autoimmune antibodies, such as antinuclear and antiphospholipid antibodies, were negative. WBC, white blood cell; Hb, hemoglobin; Plt, platelets; PT-INR, prothrombin time-international normalized ratio; APTT, activated partial thromboplastin time; APA, antiphospholipid antibodies; WT1mRNA, Wilms’ tumor 1 messenger ribonucleic acid; ANA, antinuclear antibody

Test	Value	Normal range	Test	Value	Normal range
WBC	4000/μL	3100-8400	APA	(-)	
Hb	14.6 g/dL	11.4-14.6	Protein C	94%	70-150
Plt	9.2 × 10^3^/μL	15-40	Protein S	65%	67-164
PT-INR	0.94	0.85-1.15	Antithrombin Ⅲ	96%	80-130
APTT	28.4 sec	25-40	WT1mRNA	1300 copies/μgRNA	<50 copies/μgRNA
D-dimer	1.08 μg/mL	<1.0	ANA	(-)	

Considering the large Eustachian valve with a large PFO, suspected a hypercoagulable state by no CR of AMoL, and no other embolic sources, paradoxical cerebral embolism was the most presumable diagnosis. Since he has a large Eustachian valve, which is associated with a PFO closure procedure failure [4], we decided to prioritize anticoagulant therapy. After re-starting DOAC for secondary prevention in accordance with DVT [5], stroke recurrence did not recur for over one year. In the present case, PFO closure would be considered if ischemic stroke recurrence or hemorrhagic events occur under anticoagulation therapy.

## Discussion

This case was repeated paradoxical cerebral embolism due to a large Eustachian valve and a large PFO under the background of thrombosis due to a no CR of AMoL. Although no obvious DVT was detected in this case, the exclusion of other embolic sources led us to consider paradoxical cerebral embolism from thrombus formation in the EV under a hypercoagulable state by AMoL. 

The eustachian valve is a remnant structure. Patients with an Eustachian valve have a high prevalence of PFO, and a large Eustachian valve enhances right-to-left shunt (RLS) in PFO patients by changing the venous flow toward PFO [6]. Moreover, the previous cases demonstrated that a hypercoagulable state can provoke thrombus formation in the Eustachian valve and cause paradoxical embolism [2]. Therefore, the large Eustachian valve itself could be the risk of paradoxical embolism. In the present case, the large Eustachian valve and a large PFO were considered high risk of PFO-related stroke with large vessel occlusion.

Patients with hematologic malignancies often present with a hypercoagulable state in the absence of active thrombosis [7]. In the present case, WT1mRNA was continuously high from the past stroke, suggesting no molecular CR [3]. Although there were no abnormalities in coagulation status in the laboratory data, AMoL in the present case would have the risk of thrombosis. In addition, the present case illustrates that if there is a thrombogenic environment, such as a large Eustachian valve, the thrombosis risk would be enhanced even in the clinical CR of AMoL.

In this case, by excluding other etiologies such as aortic or carotid atherosclerosis, pulmonary shunt, and AF, each of the large Eustachian valves and AMoL were presumed to be a possible cause of embolic sources. These embolic sources may be easily overlooked in the usual workup of embolic sources. The diagnosis of covert AF is especially difficult unless prolonged ECG monitoring, including ILR. Therefore, physicians should carefully review these backgrounds in case of recurrent cryptogenic stroke and consider ILR implantation for the etiology diagnosis.

## Conclusions

The present case was diagnosed with recurrent paradoxical cerebral embolism due to the large Eustachian valve and PFO under no CR of AMoL. This case illustrated that based on the hematologic malignancy, venous thrombus formation could occur in a large Eustachian valve and subsequently cause paradoxical cerebral embolism. When there are multiple suspicious embolic sources, the exclusion of AF by ILR is also useful for estimating or excluding the mechanism of cryptogenic stroke.
